# Human cervicovaginal fluid biomarkers to predict term and preterm labor

**DOI:** 10.3389/fphys.2015.00151

**Published:** 2015-05-13

**Authors:** Yujing J. Heng, Stella Liong, Michael Permezel, Gregory E. Rice, Megan K. W. Di Quinzio, Harry M. Georgiou

**Affiliations:** ^1^Department of Pathology, Harvard Medical School and Beth Israel Deaconess Medical CenterBoston, MA, USA; ^2^Department of Obstetrics and Gynaecology, University of MelbourneMelbourne, VIC, Australia; ^3^Mercy Perinatal Research Centre, Mercy Hospital for WomenHeidelberg, VIC, Australia; ^4^University of Queensland Centre for Clinical ResearchHerston, QLD, Australia

**Keywords:** cervicovaginal fluid, preterm birth, preterm labor, predictive biomarkers, pregnancy, IL-1 receptor antagonist, thioredoxin, vitamin D binding protein

## Abstract

Preterm birth (PTB; birth before 37 completed weeks of gestation) remains the major cause of neonatal morbidity and mortality. The current generation of biomarkers predictive of PTB have limited utility. In pregnancy, the human cervicovaginal fluid (CVF) proteome is a reflection of the local biochemical milieu and is influenced by the physical changes occurring in the vagina, cervix and adjacent overlying fetal membranes. Term and preterm labor (PTL) share common pathways of cervical ripening, myometrial activation and fetal membranes rupture leading to birth. We therefore hypothesize that CVF biomarkers predictive of labor may be similar in both the term and preterm labor setting. In this review, we summarize some of the existing published literature as well as our team's breadth of work utilizing the CVF for the discovery and validation of putative CVF biomarkers predictive of human labor. Our team established an efficient method for collecting serial CVF samples for optimal 2-dimensional gel electrophoresis resolution and analysis. We first embarked on CVF biomarker discovery for the prediction of spontaneous onset of term labor using 2D-electrophoresis and solution array multiple analyte profiling. 2D-electrophoretic analyses were subsequently performed on CVF samples associated with PTB. Several proteins have been successfully validated and demonstrate that these biomarkers are associated with term and PTL and may be predictive of both term and PTL. In addition, the measurement of these putative biomarkers was found to be robust to the influences of vaginal microflora and/or semen. The future development of a multiple biomarker bed-side test would help improve the prediction of PTB and the clinical management of patients.

## Introduction

A human pregnancy has a gestational period between 37 and 42 weeks. Births before 37 completed weeks of gestation are classified as preterm. Preterm birth (PTB) remains a major obstetric complication that leads to an increased risk of adverse neonatal morbidity and mortality (March of Dimes Foundation, 2009). Currently, the ability to predict PTB is disappointing. Biomarkers that can predict PTB with improved sensitivity and specificity are required. In pregnancy, the human cervicovaginal fluid (CVF) proteome reflects the biochemical milieu and physiological changes of the vagina, cervix and adjacent overlying fetal membranes. Although the initiating triggers may differ, the physiology of human parturition at term and preterm gestations share the common pathways of cervical ripening, myometrial activation and membrane rupture. In this review, we describe our team's breadth of work utilizing the CVF for the discovery and validation of biomarkers predictive of human labor.

## Physiology of human labor

Human parturition is characterized by regular and painful uterine contractions that increase in duration, intensity, and frequency. With each successive contraction, the fetus progressively descends and exerts pressure on the cervix and overlying fetal membranes. This promotes remodeling of the cervix leading to effacement and dilatation (Rechberger and Woessner, 1993; Ludmir and Sehdev, 2000), weakening and rupture of fetal membranes (Moore et al., 2006; Kendal-Wright, 2007) and the subsequent delivery of the neonate. In general, the spontaneous onset of uterine contractions and cervical remodeling precede fetal membrane rupture (Norwitz et al., 1999). However, the spontaneous rupture of the fetal membranes can also occur in the absence of uterine contractions and/or cervical dilatation at term and preterm gestations, i.e., pre-labor rupture of membranes (PROM). PROM may arise from a different physiological pathway than that of spontaneous term and preterm labor (PTL; Fortunato and Menon, 2001).

Parturition is a complex systemic physiological event involving multiple reproductive or gestational tissues (fetal membranes, uterus, cervix and vagina) and various biochemical and physical pathways including: hormonal regulation [e.g., activation of the fetal hypothalamic-pituitary-adrenal axis (Challis et al., 2000), progesterone (Mesiano et al., 2002; Challis et al., 2003), estrogen (Adachi and Oku, 1995) and prostaglandin (Mitchell et al., 1995; Romero et al., 1996)]; inflammation (Liggins, 1989; Romero et al., 1989; Thomson et al., 1999; Ledingham et al., 2000, 2001; Rauk and Chiao, 2000; Haddad et al., 2006; Osman et al., 2006; Heng et al., 2014a); mechanical stretch (Kloeck and Jung, 1973; Maehara et al., 1996; Maradny et al., 1996; Hall et al., 1997; Lyall et al., 2002; Loudon et al., 2004; Terzidou et al., 2005; Turton et al., 2009); oxidative stress (Yin and Zhen, 1995; Yoshida et al., 2001; Ekerhovd et al., 2002; Vaisanen-Tommiska et al., 2003; Burton and Jauniaux, 2010; Heng et al., 2010a; Chai et al., 2012); apoptosis (Gearing et al., 1994; Menon et al., 2002; Moore et al., 2002, 2003; El Khwad et al., 2006; Reti et al., 2007); lipid metabolism (Skannal et al., 1997; Slater et al., 2004; Farina et al., 2007); and extracellular matrix (ECM) remodeling (Matrisian, 1992; Myers and Nathanielsz, 1993; Vadillo-Ortega et al., 1995; Xu et al., 2002; Menon and Fortunato, 2004; Timmons and Mahendroo, 2007; Nishihara et al., 2008).

## Preterm birth

Globally, approximately 15 million babies are born prematurely each year. PTB rates are highest (15% or higher) in developing nations such as Pakistan, Indonesia and sub-Saharan Africa (World Health Organisation, 2012). In 2010, there were 22,952 PTB out of 297,357 live births (8.3%) in Australia (AIHW et al., 2012). PTB rates in the United States have reached 12% and have remained consistently high between 1990 and 2010 (Kuehn, 2012; World Health Organisation, 2012; Martin and Osterman, 2013). The rising incidence of PTB, especially late PTB between 34 and 36 weeks of gestation, is thought to be the result of obstetric complications and the increase in multifetal pregnancies associated with modern fertility treatments (Ananth and Vintzileos, 2006).

PTB contributes to the 3.1 million neonatal deaths worldwide annually (35%) (World Health Organisation, 2012). The survival rate of premature babies correlates with advancing gestational age. In England the survival rate for preterm neonates born at 23 weeks is 19% with the rate increasing to 77% if birth occurs at 26 weeks (Costeloe et al., 2012). Although advances in perinatal care have improved survival rates, cognitive impairment and other life-long disabilities associated with preterm birth remain significant (Lorenz, 2001; Greenough et al., 2005; Field et al., 2008; Moster et al., 2008; Johnson et al., 2009). It is not surprising that PTB carries an immense financial burden particularly in developed countries where advanced medical resources are widely available. Premature babies require a longer hospital stay, have medical complications necessitating multiple hospital admissions and the use of medical equipment and operative procedures (St. John et al., 2000; Petrou, 2005; Gilbert, 2006). The annual economic cost of PTB is estimated at USD$26 billion in the United States (Institute of Medicine, 2007) and £3 billion in the United Kingdom (Mangham et al., 2009).

PTB can be categorized as either spontaneous or iatrogenic. About 75% of PTB are spontaneous, resulting from spontaneous PTL with intact membranes (45%) or spontaneous preterm pre-labor rupture of fetal membranes (preterm PROM; 30%). The remaining 25% of PTB are iatrogenic due to maternal (e.g., diabetes), placental (e.g., pre-eclampsia) or fetal disorders (e.g., intra-uterine growth restriction) that necessitate delivery of a compromised fetus and/or mother (Moutquin, 2003; Steer, 2006).

The etiology of PTB is multifactorial. Multiple pregnancy, previous PTB, ethnicity (especially of African descent, Zhang and Savitz, 1992; Adams et al., 2000) and second- or third-trimester vaginal bleeding are most consistently associated with PTB. Other factors associated with the initiation of PTL include: poor maternal sociobiological variables (stress, malnutrition, smoking, low socioeconomic status, heavy physical work and substance abuse); fetal physiological stress (malformation, intra-uterine growth restriction); infection; preterm PROM; cervical dysfunction; placental abruption; uterine over distension (polyhydramnios or multifetal gestation); and genetic predisposition (familial factors and genetic mutation) (Hall et al., 1997; Mercer et al., 1999; Genç et al., 2002; Lockwood, 2003; Goffinet, 2005; Oyelese and Ananth, 2006; Steer, 2006; Holst and Garnier, 2008).

The treatment of PTL involves the use of tocolytic agents (e.g., β-adrenergic receptor agonists such as ritodrine or calcium channel blockers such as nifedipine) to inhibit uterine contractions (Barden et al., 1980; Ulmsten et al., 1980). Tocolytics may stop contractions temporarily but rarely prevent PTB (Goldenberg, 2002). Thus, the current application of tocolytics is largely directed at prolonging the pregnancy for up to 48 h to augment fetal lung maturity after maternal corticosteroid administration and arrange transport of the woman to a tertiary hospital with appropriate neonatal facilities (Lyndon, 2006).

The prevention of PTB in women with known risk factors may be divided into two approaches: (i) to reduce clinical risk factors by antenatal education (e.g., optimal nutrition, McGregor et al., 2001; Carlson et al., 2013) and reducing physical and emotional stress and substance abuse; and (ii) optimize appropriate use of pharmacological agents such as the use of progesterone. Progesterone has been clinically trialed to prevent PTB with encouraging results (reviewed by Iams, 2014). The prevention of PTB is more challenging for women in their first pregnancy who have no known risk factors. For all women, screening programs to detect early cervical change by ultrasonography are of limited benefit. Alternatively, diagnostic screening tools (biochemical markers) to reliably predict the onset of (preterm) labor (Goldenberg, 2002) are required. The prognostic value of the current biochemical markers to predict PTB is disappointing. The relatively poor sensitivity and positive predictive value of these tests often lead to costly and unnecessary interventions.

## Predicting human labor

Given the challenges of preventing PTB, the ability to reliably predict who will deliver preterm may aid in the clinical management of asymptomatic or symptomatic women (i.e., in threatened PTL with uterine contractions without cervical dilatation). The accurate prediction of PTL is difficult due to the multifactorial etiology of PTB as various biochemical triggers may result in different clinical presentations of early onset labor. Therefore, a single predictor of PTB may yield varying utility in symptomatic women compared to asymptomatic women without or with risk factors for PTL (e.g., previous history of PTB or preterm PROM, multifetal gestation, cervical incompetence etc.).

### Risk scoring

Risk scoring is a quantitative approach to identify women as low-, medium-, or high-risk for PTB within the obstetric population (Krupa et al., 2006). A combined Australia and New Zealand cohort study of nulliparous women reported maternal demographics (ethnicity, low body mass index, smoking, drug use, and anxiety) and obstetric characteristics (>3 months to conceive, shortened cervical length, gestational diabetes, mild gestational hypertension and preeclampsia) as risk factors for spontaneous PTB and/or preterm PROM (Dekker et al., 2012). Other factors associated with a higher risk of PTB include multifetal gestation (Martin et al., 2012), bacterial vaginosis (Guaschino et al., 2006) and previous cervical cone biopsy (Raio et al., 1997). However the use of risk scoring alone is often unreliable as more than 50% of women who experience PTB have no recognizable risk factor (Mercer et al., 1999; Shankar et al., 2005; Dekker et al., 2012).

### Cervical length

Transvaginal ultrasonography was introduced in the 1980s as a screening tool to assess the cervical morphology during pregnancy (measurement of cervical length and evaluate the dilated internal cervical os, Okitsu et al., 1992; Leitich, 2005). It is a more reliable and reproducible method than digital cervical examination to predict PTB (Krupa et al., 2006). A study by Iams et al. (1996) reported increasing risk of PTB (<35 weeks of gestation) in asymptomatic women as cervical length deceases. Systematic reviews of asymptomatic women (at <24 weeks of gestation) found that a cervical length <25 mm is associated with PTB before 35 weeks of gestation, with positive likelihood ratios (LR+) ranging from 2.8 to 6.3 (Honest et al., 2003; Crane and Hutchens, 2008); and that a range of cut-off values for cervical length (25–35 mm) achieved 33–54% sensitivity and 73–91% specificity to predict PTB (Leitich et al., 1999a). The low sensitivity afforded by cervical ultrasonography limits this method as a routine screening test in low-risk asymptomatic women (Resnik, 2005).

In symptomatic women, a cervical length <15 mm yields a LR+ of 8.7 for preterm delivery within 7 days, compared to a LR+ of 0.4 when the cervical length is ≥15 mm (Gomez et al., 2005). Berghella et al. (2013) found that knowledge of cervical length was associated with a non-significant decrease (22.3%) in the incidence of PTB before 37 weeks in symptomatic women, compared to when the cervical length was not known (34.7%). Thus, the authors did not recommend the use of transvaginal ultrasonography as a routine screening tool.

### Biochemical markers

Biological fluids including amniotic fluid, urine, serum/plasma/whole blood, saliva and CVF provide rich sources of proteins and molecules which are significantly altered in response to different biological states (Darne et al., 1987; Romero et al., 1989; Agrez et al., 1999; Goldenberg et al., 2001; Di Quinzio et al., 2008; Heng et al., 2014a). In symptomatic women, fetal fibronectin (fFN) and phosphorylated insulin-like growth factor binding protein-1 (phIGFBP1) bedside test kits utilizing CVF are currently used to predict PTB. However, no current single biomarker can positively predict PTB reliably in either asymptomatic or symptomatic women.

#### Fetal fibronectin

The fFN test is more commonly used to predict PTB in symptomatic women with threatened PTL but has also been investigated in asymptomatic pregnant women. fFN is a 450 kDa glycoprotein secreted by trophoblasts in the fetal membranes which maintains the integrity of the ECM at the interface between the fetal chorion and maternal decidua (Sibille et al., 1986). It is generally not detectable in the CVF of women after 16 to 20 weeks of gestation. The detection of fFN between 22 and 34 weeks of gestation may signify the premature detachment of the fetal chorion from the maternal decidua (Sibille et al., 1986; Lockwood et al., 1991). The pioneering study by Lockwood et al. (1991) described the clinical utility of fFN to predict spontaneous PTB in symptomatic women (*n* = 117 women) with 81.7% sensitivity, 82.5% specificity, 83.1% positive predictive value (PPV), and 81.0% negative predictive value (NPV), based on a fFN threshold concentration of ≥50 ng/mL.

In *asymptomatic* women, fFN has low sensitivity (20–29%) and poor PPV (17–25%) to predict PTB at <34 weeks of gestation; but the NPV remains high (96–97%) (Goldenberg et al., 1996). A subsequent meta-analysis by Leitich et al. (1999b) of asymptomatic women reported a sensitivity of 22% but comparable specificity of 97% of fFN to predict spontaneous PTB within seven days. By contrast, in *symptomatic* women, meta-analyses performed by Sanchez-Ramos et al. (2009) and Boots et al. (2014) reported improved predictive utility of fFN with 75–76% sensitivity and 79–82% specificity to predict PTB within 7 days.

Fetal fibronectin testing may not be feasible in up to 50% of women due to recent vaginal digital examination, unprotected sexual intercourse, vaginal bleeding or amniotic fluid contamination from the rupture of fetal membranes as these may cause a false positive result (Sadovsky and Friedman, 1992; Shimoya et al., 1998). Thus, the poor sensitivity, relatively low patient eligibility and false positive results all limit fFN as a screening tool for PTB. It is now generally accepted that the fFN test is most clinically useful for its high NPV to predict PTB within seven to 14 days of sampling (Goldenberg, 2002; Honest et al., 2002; Ramsey and Andrews, 2003). A recent proposal to combine cervical length and fFN to screen symptomatic (Hincz et al., 2002; Schmitz et al., 2006; Dutta and Norman, 2010; DeFranco et al., 2013) and asymptomatic women (Bolt et al., 2011; Fox et al., 2012) appears to result in greater sensitivity and PPV, compared to fFN testing alone, to predict PTB. Further studies will determine whether this combined screening approach can be informative for the clinical management of women in the future.

#### Phosphorylated insulin-like growth factor binding protein-1

The IGFBP1 test is another commonly used predictive test for PTL. IGFBP1 is a 25kDa protein that is secreted by maternal decidual cells as a highly phosphorylated isoform, phIGFBP1 (Rutanen et al., 1985; Westwood et al., 1994; Martina et al., 1997). The non-phosphorylated form of IGFBP1 is predominately present in the amniotic fluid (Nuutila et al., 1999). The concentration of IGFBP1 increases at the beginning of the second trimester in amniotic fluid and decidua when the amnion fuses with the choriodecidua (Wathen et al., 1993) and the degree of phosphorylation increases until late pregnancy (Koistinen et al., 1993). Like fFN, the detection of phIGFBP1 in the CVF indicates a disruption of the choriodecidual interface.

phIGFBP1 was first evaluated to diagnose preterm PROM with high sensitivity and a positive test was associated with a 6.9-fold increased risk of PTB (Rutanen et al., 1996). Kekki et al. (2001) conducted a study of 63 symptomatic women where subjects with >10 mg/L of cervical phIGFBP1 had a ten-fold higher risk of PTB. phIGFBP1 predicts spontaneous PTB in women with threatened PTL with 69–70% sensitivity, 74–90% specificity, 48–50% PPV and 88–96% NPV (Paternoster et al., 2007; Tanir et al., 2009); whilst a meta-analysis reported that phIGFBP1 predicts PTB in asymptomatic women with a pooled 33% sensitivity, 79% specificity, LR+ of 1.6 and a LR− of 0.8 (Conde-Agudelo et al., 2011).

The phIGFBP1 test has a comparable NPV to the fFN test in predicting spontaneous PTB within 7 days in symptomatic women (phIGFBP1 92% vs. fFN 97%) (Ting et al., 2007). In asymptomatic women, phIGFBP1 and fFN have significantly different PPVs (phIGFBP1, 0% vs. fFN, 67%) but similar NPVs (phIGFBP1, 70% vs. fFN, 79%) for predicting PTB (Khambay et al., 2012). phIGFBP1 is not affected by the presence of seminal fluid or vaginal bleeding. These studies indicate that, like fFN, the clinical utility of the phIGFBP1 test, is as a reliable negative predictor of PTB but thus far no systematic meta-analyses on the predictive utility of phIGFBP1 have been performed.

#### Other biochemical markers

Given the poor sensitivity of the fFN and phIGFBP1 tests to predict spontaneous PTB in the asymptomatic pregnant population, researchers have investigated alternative biomarkers associated with PTL and assessed their predictive utility. The evaluation of these putative biomarkers indicates that no single biomarker is superior in predicting spontaneous PTB (Wei et al., 2010; Conde-Agudelo et al., 2011; Menon et al., 2011; Bhat et al., 2013), perhaps reflecting the different aetiologies associated PTL. The involvement of multiple biochemical pathways associated with parturition presents a challenge to identify a single biomarker that reliably predicts human labor. Furthermore, reproducibility is a common problem encountered by different biomarker validation studies. Experimental design including the CVF sampling method, patient selection/exclusion criteria (e.g., intact vs. ruptured membranes), sample selection/exclusion criteria, varying sample sizes and different statistical approaches used in predictive modeling analyses can all affect the outcome of results. The importance of a well-designed study with strict patient selection criteria is crucial for a fair and accurate comparison of putative biomarkers to predict spontaneous PTB.

### Summary of commonly used screening tests

The advantages and disadvantages of the most commonly used tests to predict PTL are summarized in Table 1. While risk scoring can be performed essentially free of cost, extensive demographic and medical history data relating to the current and previous pregnancies is essential. Transvaginal ultrasonography can often be informative but relies on the availability of expensive ultrasound equipment and a skilled ultrasonographer to interpret the information. At best, ultrasound measurements of the cervix can serve as an adjunct to other screening tests. The biochemical tests continue to be unreasonably expensive but can provide valuable reassurance (negative likelihood) particularly for the symptomatic woman from a remote community where neonatal intensive care facilities are not available.

**Table 1:** **Summary of commonly used tests to predict preterm labor**.

**Method**	**Approach**	**Advantages**	**Disadvantages**
Risk scoring	Computer-based algorithm to determine risk	Essentially “no cost” Fairly rapid and easy to perform	Requires extensive demographic and medical history information Poor predictive utility
Cevical length	Transvaginal ultrasonography	Can be incorporated during routine antenatal examination Essentially “no additional cost” Provides useful information on the state of the cervix Fairly rapid to perform	Expensive ultrasound equipment required Requires skilled ultrasonographer Limited predictive utility but may be a useful adjunct to biochemical tests
Biochemical tests	“Dip-stick” tests Quikcheck (fFN) Actim partus (phIGFBP1)	Rapid and easy to perform Good negative predictive value	Moderately expensive analyzer Expensive single test May be influenced by contaminants (blood, amniotic fluid, semen) Semi-quantitative Predetermined threshold cut-off Poor positive predictive value
	Immunoassay Rapid TLi_IQ_ (fFN)	Rapid and easy to perform Good negative predictive value	Moderately expensive analyzer Expensive single test May be influenced by contaminants (blood, amniotic fluid, semen) Semi-quantitative Predetermined threshold cut-off Poor positive predictive value
	Immunoassay Rapid 10Q (fFN)	Rapid and easy to perform Accurate quantification Ability to choose the desired threshold cut-off Good negative predictive value	Moderately expensive analyzer Expensive single test May be influenced by contaminants (blood, amniotic fluid, semen) Poor positive predictive value

## Cervicovaginal fluid biomarkers of human labor

High throughput proteomic techniques have expanded biological studies from the biochemical analysis of single proteins to proteome-wide investigations that have allowed the direct comparison of novel or differentially expressed proteins to be made between disease and non-disease states (Colantonio and Chan, 2005; Monti et al., 2005). Extensive descriptive proteomic studies of urine (Coon et al., 2008; Zürbig et al., 2009), plasma, saliva (Yan et al., 2009), lung tissues, bronchoalveolar lavage (Marko-Varga et al., 2005), amnion, amniotic fluid (Nilsson et al., 2004; Park et al., 2006; Michaels et al., 2007), placenta (Butt et al., 2006) and gynecological carcinomas including the ovary (Alaiya et al., 1999), vagina (Hellman et al., 2004) and cervix (Bae et al., 2005) have been described. These proteomic maps are catalogs of the protein complement in a specific tissue or body fluid, representing the presence and the relative abundance of proteins at a given point in time (Pennington et al., 1997).

Human CVF is a rich source of proteins and other metabolites ideal for the discovery of biomarkers associated with gynecological and pregnancy complications. Human labor involves cervical remodeling, myometrial activation and rupture of the fetal membranes. Given that the CVF is largely a product derived from these gestational tissues, the precise biochemical mechanisms involved and the timing of these dynamic processes during labor may be reflected in the CVF. Historically, CVF proteins have been selectively targeted and studied to predict labor, such as fFN (Lockwood et al., 1991), IGFBP1 (Kekki et al., 2001), defensins and lactoferrin (Goldenberg et al., 2001), sialidase (Andrews et al., 1999; Goldenberg et al., 2001), granulocyte elastase (Nakai et al., 2005), human chorionic gonadotropin (Sanchez-Ramos et al., 2003; Garshasbi et al., 2004), interleukin (IL) 1beta (Tanaka et al., 1998), IL6 (Rizzo et al., 1996; Coleman et al., 2001; Grenache et al., 2004; Holst et al., 2005; Woodworth et al., 2007), IL8 (Rizzo et al., 1998; Tanaka et al., 1998; Holst et al., 2005), IL18 (Jacobsson et al., 2003), interleukin-1 receptor antagonist (IL1RN) (Rizzo et al., 1996) and tumor necrosis factor (Rizzo et al., 1996; Grenache et al., 2004).

### Cervicovaginal fluid proteome

Our research group was the first to publish the CVF proteome map of pregnant women utilizing 2-dimensional-polyacrylamide gel electrophoresis (2D-PAGE) and mass spectrometry (matrix-assisted laser desorption/ionization time of flight, MALDI-ToF, or liquid chromatography coupled to an electrospray ion-trap, LC-ESI-MS/MS). Twenty-one proteins spots were characterized, yielding 15 different proteins involved in the regulation of oxidative stress defense, proteolysis and inflammation (Di Quinzio et al., 2007). A comprehensive CVF proteomic study by Dasari et al. (2007) was published a few months later. They identified 150 unique proteins in second trimester pregnant women using LC-MS/MS and gel electrophoresis. The CVF in these two studies was collected using either rayon (Di Quinzio et al., 2007) or Dacron® swabs (Dasari et al., 2007) and protein was later extracted into a solubilization buffer. Four research papers on the CVF proteome were subsequently published. Tang et al. (2007) identified and characterized 147 proteins by 2D-PAGE and MALDI-ToF/ToF in the vaginal lavage from non-pregnant, non-infected women and validated selected proteins by immuno-blotting. Shaw and co-workers (Shaw et al., 2007) collected CVF from healthy non-pregnant women by inserting a 5 × 5 cm sterile gauze into the vagina and extracting the fluid into 10 mL of PBS; 685 proteins were identified using 1D-PAGE and strong cation exchange chromatography, followed by LC-MS/MS. Pereira et al. (2007) identified 205 proteins using multidimensional protein identification technology (MudPIT) from CVF collected using Dacron swabs from asymptomatic controls and symptomatic women admitted in threatened PTL. Klein et al. (2008) collected CVF samples from women admitted with symptoms of threatened PTL using vaginal wash (for 2D-PAGE) and Dacron swabs (for LC-MS/MS) and identified 39 unique proteins.

Whilst these studies (Dasari et al., 2007; Pereira et al., 2007; Shaw et al., 2007; Tang et al., 2007; Klein et al., 2008) produced comprehensive proteomic “maps” or “fingerprints,” it is unclear what proportion of the identified proteins were common to all women. Work from our laboratory has focused on common CVF proteins in pregnant women (Di Quinzio et al., 2007, 2008; Heng et al., 2010b; Liong et al., 2013a,b, 2015). For example, in our 2007 paper, 2D-PAGE gels were compared between five pregnant women at term. Although the number of protein spots visualized in each gel varied from 361 to 484, only about one third of the protein spots (*n* = 157) were common to all five women (Di Quinzio et al., 2007). This may be attributed to individual biological variation and the non-sterile vaginal environment. Zegels et al. (2009) analyzed CVF samples obtained via colposcopy and compared their findings with published CVF proteomic studies. The large variation of identified proteins amongst these studies were attributed to biological factors (menstruation, age, infection, unprotected sexual intercourse, use of contraceptives, pregnancy etc.), and different sampling and analytical methods. The creation of a core CVF proteome reference library may facilitate future research studies.

### Proteomic studies of preterm birth

Proteomic technologies have been applied to amniotic fluid in search of biomarkers of intra-amniotic infection (Gravett et al., 2004; Buhimschi et al., 2005; Rüetschi et al., 2005; Bujold et al., 2008), PTB (Romero et al., 2010; Fotopoulou et al., 2012) and preterm PROM (Vuadens et al., 2003; Wang et al., 2011; Tambor et al., 2012); in the placentae for preterm PROM (Chang et al., 2013), in the serum for PTB (Pereira et al., 2010; Esplin et al., 2011) and recently, in leukocyte lysates for PTB (Heng et al., 2015). Studies of the CVF proteome in association with PTB have been reviewed by Zegels et al. (2010). One of the first CVF discovery studies for PTB identified an increased expression of bactenecin-1 in the CVF of sheep after fetal glucocorticoid-induced PTL (Young et al., 2007). There have been eight subsequent discovery-based studies of human CVF, including five from our group, that have explored proteomic changes in association with spontaneous term labor (Di Quinzio et al., 2008; Heng et al., 2010b), PTL (Pereira et al., 2007; Shah et al., 2009; Liong et al., 2013a, 2015; Lo et al., 2014) or preterm PROM (Liong et al., 2013b).

Pereira et al. (2007) compared the CVF proteome between three groups of women: symptomatic women admitted with threatened PTL who delivered preterm, symptomatic women in “false” labor who delivered at term and asymptomatic gestation-matched controls. They reported 28 (using MudPIT analysis) and 17 (using two-dimensional difference in gel electrophoresis, 2D-DIGE) significantly altered proteins, including annexin A3, annexin V, cystatin A, profilin 1, group-specific component (vitamin D binding protein) and IGFBP1 fragments (16 and 11 kDa). Western blot validated the differential expression of calgranulin A and B, annexin V, profilin 1, and IGFBP1.

Shah et al. (2009) identified 236 proteins common to endocervical columnar epithelial and vaginal mucosal human cell lines by using stable isotope labeling by amino acids in cell culture methodology. Fifteen proteins were selected as candidate PTB biomarkers and the authors created a multiple reaction monitoring assay to screen CVF samples (collected using Dacron swabs). Three proteins, desmoplakin isoform 1, stratifin, and thrombospondin 1 precursor were significantly elevated in five asymptomatic women who had spontaneous PTB between 28 and 32 weeks of gestation compared to five controls who delivered at term.

Lo et al. (2014) identified 15 CVF proteins differentially expressed in asymptomatic women with recurrent PTB compared to gestation-matched controls. CVF collected in asymptomatic women (using Dacron swabs) were within 2–14 days before spontaneous PTB. Cysteine-rich secretory protein 3, heat shock protein beta 1, psoriasin and alpha enolase 1 demonstrated the largest difference in spectral counts between controls and PTB. However, the authors could not confirm these findings using enzyme-linked immunosorbent assays (ELISA) in an independent cohort of 20 women.

The remainder of this review will summarize our team's breadth of work utilizing the CVF to identify biomarkers associated with human labor. We describe the recruitment of patients and the study cohorts, the methods used to identify and quantify the differentially expressed proteins, the validation of candidate biomarkers, the influence of vaginal microflora and semen, and the challenges encountered in developing multiple biomarker models to predict term and PTB.

## Biomarkers predictive of human labor

### Patient groups and cervicovaginal fluid collection

CVF samples were collected using a rayon double-tip swab and processed using our published protocol (Di Quinzio et al., 2007). At the time of sampling, a high vaginal swab was taken for microbiology culture and assessment, and the participants were asked whether they had (1) unprotected sexual intercourse in the preceding 48 h; (2) recent vaginal bleeding; (3) an internal ultrasound or internal vaginal examination in the preceding 6 h; and (4) antibiotic treatment. Obstetric, medical and demographic data were also collected.

The CVF sample bank consisted of samples collected from three groups of pregnant women:

1. Term labor: healthy, multiparous women with a singleton pregnancy were recruited and sampled weekly starting at 36 weeks of gestation until delivery. An in-labor sample (i.e., spontaneous onset, intact membranes, cervical dilatation >40 mm) was collected, when possible.
2. At-risk of PTL: participants with at least one identified clinical risk factor for preterm birth were recruited. The clinical risk factors included multifetal pregnancy, history of PTL or preterm PROM, heavy smoker, uterine anomaly (e.g., bicornuate uterus) and history of a cervical cone biopsy and/or a shortened cervix (<25 mm based on ultrasonographic determination). CVF samples were collected serially from participants with intact fetal membranes at four weekly intervals, starting from 22 to 25 weeks of gestation until 36 weeks of gestation. Participants were stratified based on birthing outcomes: spontaneous PTL, spontaneous preterm PROM and spontaneous term labor.
3. Threatened PTL: women admitted with symptoms of preterm labor between 22 and 36 weeks of gestation were recruited and a single CVF sample was collected at the time of admission.

CVF was not collected if rupture of the fetal membranes was confirmed owing to the likely contamination of the CVF with amniotic fluid. Participants were not recruited if the woman had experienced an internal vaginal examination or transvaginal ultrasound in the preceding 6 h, was receiving progesterone pessary treatment, had a cervical cerclage *in situ*, experienced heavy antepartum hemorrhage, pre-eclampsia, intra-uterine growth restriction, or hypertension. Women presenting with suspected chorioamnionitis were excluded as well as those subsequently diagnosed with bacterial vaginosis.

### Methods: biomarker discovery using 2D-PAGE and 2D-DIGE

2D electrophoretic methodologies (2D-PAGE and 2D-DIGE) involve the combination of two orthogonal electrophoretic separation processes on a gel at right angles to provide high resolution separation of complex protein solutions. Proteins are first separated in the first dimension based on their isoelectric point followed by molecular weight separation in the second dimension. While 2D-PAGE allows the comparison between individual samples, 2D-DIGE is designed to compare groups of pooled samples (Unlu et al., 1997). Following electrophoresis, gels are stained with silver, dyes or fluorophores for visualization and imaging. Each protein spot on a 2D electrophoresis gel generally corresponds to a single protein or an isoform of a protein. Using computer-assisted image analysis, differentially expressed proteins and/or proteins of interest can be subsequently excised from the gel and characterized using mass spectrometry.

Detailed 2D-PAGE and 2D-DIGE protocols using CVF for putative biomarker discovery have been published by our team (Di Quinzio et al., 2007, 2008; Heng et al., 2010b; Liong et al., 2013a,b).

All proteomic techniques have limitations. 2D electrophoresis can be a laborious process and in turn may cause procedural protein loss. A typical 2D electrophoresis protocol may result in up to 80% of protein loss and therefore spot intensities may not be reliable indicators of protein abundance in the original sample (Zhou et al., 2005). The discovery of putative biomarkers using 2D electrophoresis is restricted to “visible” and sufficiently abundant protein spots. Low abundance proteins may not be successfully identified or investigated and 2D electrophoresis analysis may be affected by peptide fragmentation resulting in two or more spots of the same protein. Often, low abundance proteins can only be visualized after pre fractionation and removal of high abundance proteins. Another issue of 2D electrophoresis is the poor characterization of water-insoluble proteins and transmembrane proteins (e.g., exosomes, membrane fragments and their associated proteins). These proteins remain insoluble due to the use of non-ionic chaotropic detergents (e.g., urea and thiourea) to re-solubilize proteins after sample precipitation and low ionic strength detergents (to generate high electric fields) during IEF separation (Santoni et al., 2000). Despite the limitations of 2D electrophoresis methods, they remain a useful tool for biomarker discovery but these limitations highlight the importance of performing appropriate independent validation studies.

### Methods: biomarker validation using immunobased techniques

Putative biomarkers associated with labor and identified by 2D electrophoresis were validated using three techniques:

1. Western blot (protein immunoblot) is a semi-quantitative method used to detect specific proteins in a sample (Burnette, 1981; Gorr and Vogel, 2015). In order to compare protein expression between samples, the target protein is often normalized against a “housekeeping” protein such as actin or tubulin. However, as CVF lacks a housekeeping protein, we normalized against the protein load of the sample.
2. ELISA can rapidly validate and quantify a target protein in a given sample (Lequin, 2005). Although ELISA methods are sensitive and specific in determining the concentration of the target protein, a significant limitation of the ELISA is that only one analyte can be quantified at a time. This can be time consuming when measuring multiple analytes.
3. Solution array multiple analyte profiling or multiplex assay combines the technologies of dual laser flow cytometry, microspheres, rapid digital signal processing and traditional immunochemistry to allow the simultaneous quantitative measurement of multiple analytes (up to 500 analytes) in a 96-well microplate format (Mandy et al., 2001; Elshal and McCoy, 2006; Houser, 2012). This method is useful for rare or volume-limited samples.

## Biomarker discovery—term parturition

2D-PAGE and candidate-based multiple analyte profiling were utilized to discover and characterize differential CVF protein biomarkers associated with spontaneous onset of term labor. In the first 2D-PAGE/MALDI-ToF study, proteins of 12-30 kDa (using 8–16% Tris/HCl gels) were analyzed from paired CVF samples collected from nine women at term. Samples were collected 26–30 days before spontaneous labor onset, 1–2 days before labor onset, and in labor prior to rupture of the fetal membranes (Di Quinzio et al., 2008). Nine proteins were differentially expressed 26–30 days before spontaneous term labor onset compared to in-labor samples [annexin A3 (ANXA3), serpin B4 (SERPINB4), copper, zinc superoxide dismutase (SOD1), cystatin A (CSTA), epidermal fatty acid-binding protein (FABP5), glutathione S-transferase pi 1 (GSTP1), interleukin-1 receptor antagonist (IL1RN), peroxiredoxin-2 (PRDX2), and thioredoxin-1 (TXN)]. ANXA3, CSTA, FABP5, SERPINB4, and TXN were also significantly altered 1–2 days prior to spontaneous labor onset compared to the 26–30 day samples.

Protein spots in the upper section of the 8–16% gel (>37 kDa region) were inadequately resolved for analysis. A subsequent 2D-PAGE study utilized 10% Tris/HCl gels and MALDI-ToF to identify proteins between 25-45 kDa that were differentially expressed in association with spontaneous term labor onset (Heng et al., 2010b). Four serial samples from nine women were collected at 14–17 days, 7–10 days, 0–3 days^1^ prior to term labor as well as samples collected in-labor prior to rupture of the fetal membranes. Albumin (ALB), ANXA3, serpin B1 (SERPINB1), serpin B3 (SERPINB3) and collagen alpha 2 type IV (COL4A2) were differentially expressed with spontaneous labor onset. It is important to highlight that differential analyses were focused on proteins common to all 2D electrophoresis gels within each study (Di Quinzio et al., 2008; Heng et al., 2010b).

## Biomarker discovery—preterm parturition

2D-DIGE and 2D-PAGE analyses were performed on CVF samples using 8–16% Tris/HCl gels from three distinct clinical cohorts who subsequently experienced PTB: (1) asymptomatic women assessed as clinically at-risk of PTB; (2) asymptomatic women who subsequently experienced preterm PROM; and (3) symptomatic women (i.e., presenting in PTL with the absence of cervical dilatation). LC-ESI-MS/MS was used to identify proteins that were differentially expressed in women who subsequently delivered preterm compared to gestation-matched women who delivered at term.

### Asymptomatic preterm parturition

Proteomic analysis of the CVF obtained from asymptomatic women 11–22 days prior to experiencing the onset of spontaneous PTL and delivery (*n* = 5) revealed 15 proteins [ALB, apolipoprotein A1 (APOA1), CSTA, FABP5, vitamin D binding protein (group-specific component, GC), gamma-glutamylcyclotransferase (GGCT), GSTP1, IL1RN, S100 calcium-binding protein A7 (S100A7), SERPINB3, serpin B4 (SERPINB4), serpin B6 (SERPINB6), SOD1, transaldolase (TALDO1), and TXN] with significantly altered expression compared to gestation-matched women who delivered at term (*n* = 10) (Liong et al., 2013a).

### Asymptomatic preterm pre-labor rupture of membranes

The current diagnosis of preterm PROM predominately relies on clinical assessments including the visual detection of amniotic fluid leaking from the cervical os and/or cervicovaginal discharge presenting with an alkaline pH (nitrazine test) (Caughey et al., 2008). The proteomic studies to date identified biomarkers that serve to confirm suspected cases of preterm PROM (Vuadens et al., 2003; Tambor et al., 2012; Chang et al., 2013) but no proteomic studies that have identified biomarkers that may predate preterm PROM. The early and accurate prediction of preterm PROM could allow for timely intervention in order to improve perinatal outcomes and reduce obstetric complications such as chorioamnionitis, neonatal sepsis or cord prolapse (Bengtson et al., 1989; Dale et al., 1989; Mercer et al., 1992)

Our group published the first prospective CVF proteomic biomarker study in women who subsequently experienced preterm PROM. Nine proteins (ANXA3, CSTA, FABP5, GC, GGCT, IL1RN, SERPINB1, SERPINB3, and TXN) were differentially expressed in asymptomatic women (*n* = 5) 6–23 days before preterm PROM and delivered preterm compared to gestation-matched controls who delivered at term (*n* = 10) (Liong et al., 2013b).

### Symptomatic preterm parturition

In the symptomatic cohort of preterm pregnant women, a single CVF sample was obtained at the time of admission to the Emergency Department. Following birthing outcomes, CVF proteomic analysis was subsequently performed on samples obtained from symptomatic women who presented with uterine contractions in the absence of cervical change and experienced spontaneous PTB (*n* = 4) and compared against gestation-matched symptomatic women who subsequently delivered at term (*n* = 8). 2D-DIGE and 2D-PAGE analyses identified six proteins (ALB, CSTA, FABP5, GC, IL1RN, and TXN) significantly differentially expressed in symptomatic women 40–54 days before spontaneous preterm birth (Liong et al., 2015).

## Candidate biomarker validation—term parturition

Based on the proteomic discovery findings described above, a selected number of putative biomarkers were validated in a large independent term cohort. In addition, a number of biomarkers previously implicated in parturition were also validated including those associated with inflammatory processes [IL-1 alpha and beta (IL1A, IL1B)], oxidative stress defense (total antioxidant capacity assay); and matrix remodeling (matrix metalloproteinases 1, 2, 3, 7, 8, and 9 (MMP1, MMP2, MMP3, MMP7, MMP8, MMP9), tissue inhibitors of metalloproteinases 1 and 2 (TIMP1, TIMP2) and cysteine proteases [cathepsins B, H, and L (CTSB, CTSH, CTSL)]. Table 2 summarizes the biomarkers that were successfully validated in large independent cohorts using ELISA, multiple analyte profiling or enzyme activity assay (Heng et al., 2008, 2010a, 2011, 2012, 2014b; Liong et al., 2013c) and the predictive utilities of some of these putative biomarkers have been published.

**Table 2:** **Validated putative biomarkers associated with term labor**.

**Function**	**Significantly increased**	**Significantly decreased**	**No significant changes**	**References**
Inflammation/anti-inflammation	IL1A, IL1B	IL1RN		Heng et al., 2008, 2014b
Oxidative stress defense		SOD1, TXN, total antioxidant capacity^*^		Heng et al., 2010a
Cysteine proteases/inhibitors		CSTA	CTSB^*^, CTSH^*^, CTSL^*^	Heng et al., 2011
Metallo proteases/inhibitors	MMP7, TIMP1, TIMP2		MMP1, MMP2, MMP3, MMP8, MMP9	Heng et al., 2012
Carrier/transport	GC			Liong et al., 2013c

*CVF samples were taken from 36 weeks of gestation and all women labored spontaneously at term*.

* *Activity assay*.

## Candidate biomarker validation—preterm parturition

Proteins differentially expressed with impending PTL in asymptomatic and symptomatic women are summarized in Table 3. For the basis of comparison, these biomarkers are compared to those associated with term labor. This is a generalized comparison as there is variation in the timing of sample collection before labor between the cohorts. As expected, not all of these novel biomarkers displayed a uniform change between the different preterm pathologies. Indeed, some biomarkers were either unique to women who experienced preterm PROM or spontaneous PTL (with intact membranes). These findings suggest that the mechanisms involved in preterm PROM (with no infection) may be different from the processes involved in spontaneous PTL with intact membranes. As the content of the CVF is reflective of the changes occurring in the surrounding tissues during cervical effacement and dilation, and the rupture of fetal membranes, these biomarkers may provide new insights into the timing and the biochemical changes prior to the onset of labor.

**Table 3:** **Putative biomarkers associated with preterm labor compared to term labor**.

	**Term Labor^+^**	**Asymptomatic Preterm Labor (PTL)^+^**	Asymptomatic Preterm Pre-labor Rupture of Membranes (PROM)^*^	**Symptomatic Preterm Labor (PTL)^+^**
	Samples beyond 36 weeks of gestation including in-labor before rupture of membranes	Two to twenty two days before spontaneous PTL (For GC, samples collected up to 120 days before spontaneous PTL)	Two to twenty days before spontaneous preterm PROM	Zero to fifty four days before spontaneous PTL
**IL1RN**	↓	↓	↓	↔
	(*n* = 240; 292 term)	(*n* = 15 preterm, *n* = 83 term)	(*n* = 5 preterm, *n* = 10 term)	(*n* = 16 preterm, *n* = 101 term)
**CSTA**	↓	↔	↓	↓
	(*n* = 247 term)	(*n* = 21 preterm, *n* = 59 term)	(*n* = 5 preterm, *n* = 10 term)	(*n* = 4 preterm, *n* = 8 term, based on 2D-PAGE only)
**TXN**	↓	↓	↔	↔
	(*n* = 163 term)	(*n* = 13 preterm, *n* = 55 term)	(*n* = 5 preterm, *n* = 10 term)	(*n* = 14 preterm, *n* = 86 term)
**SOD1**	↓	↔	↔	↔
	(*n* = 170 term)	(*n* = 24 preterm, *n* = 89 term)	(*n* = 5 preterm, *n* = 10 term, based on 2D-PAGE only)	(*n* = 4 preterm, *n* = 8 term, based 2D-PAGE only)
**ALB**	↑	↔	↑	↑
	(*n* = 36 term based on 2D-PAGE only)	(*n* = 14 preterm, *n* = 59 term)	(*n* = 5 preterm, *n* = 10 term based on 2D-PAGE only)	(*n* = 19 preterm, *n* = 98 term)
**GC**	↑	↑	↔	↑
	(*n* = 352 term)	(*n* = 89 preterm, *n* = 372 term)	(*n* = 5 preterm, *n* = 10 term)	(*n* = 16 preterm, *n* = 97 term)
**ANXA3**	↓		↑	
	(*n* = 36 term based on 2D-PAGE only)		(*n* = 5 preterm, *n* = 10 term)	
**References**	Heng et al., 2008, 2010a,b, 2011, 2014b; Liong et al., 2013c	Liong et al., 2013a,c	Liong et al., 2013b	Liong et al., 2015

+ *Validated by ELISA unless stated otherwise*.

* *Validated by Western Blot unless stated otherwise*.

## Influence of vaginal microflora and semen

An ideal diagnostic test should be robust to the high phenotypic variation among patients caused by genetic, health, lifestyle and environmental factors. Specifically, any biomarker study or diagnostic test of the CVF must ensure that the biomarker expression is resilient to the influences of latent vaginal microflora, semen and vaginal bleeding. To determine the influence of vaginal microflora on CVF proteins, upper vagina microbiology culture and assessment was performed. Medical laboratory pathology results were classified into five groups: no significant pathogen identified, Group B Streptococcus colonization, *Candida* spp. colonization, *Ureaplasma* spp. Colonization, or mixed colonization (consisting of two or more of these groups). Participants diagnosed with bacterial vaginosis were excluded from the studies. To investigate if semen or vaginal bleeding might influence CVF proteins, participants were asked if they had had unprotected sexual intercourse in the preceding 48 h or vaginal bleeding within 24 h of CVF sampling.

From the microbiology results and patient responses, validated biomarkers (ALB, CSTA, GC, IL1A, IL1B, IL1RN, SOD1, TIMP1, TXN, and total antioxidant capacity) were not influenced by common vaginal microflora or semen (Heng et al., 2008, 2010a, 2011, 2012, 2014b; Liong et al., 2013a,c, 2015). However, the influence of unprotected sexual intercourse (i.e., semen) on putative biomarkers may be limited by the information volunteered by participants. The number of women reporting vaginal bleeding was too low for analysis. Given that many CVF proteins are also present in serum, it is possible that any recent vaginal bleeding could influence the measurement of these putative CVF biomarkers. In addition, the carriage of certain gene polymorphisms (e.g., GC, IL1, and IL1RN) is associated with differing levels of protein expression (Santtila et al., 1998; Lauridsen et al., 2001; Pociot et al., 1992; Danis et al., 1995; Genç et al., 2004) and it is conceivable that the CVF expression of these proteins may be associated with specific obstetric complications.

## Multiple biomarker prediction of human parturition and its challenges

The heterogeneity of spontaneous PTB and the various clinical presentations (asymptomatic or symptomatic) provide significant challenges for the identification of a single biochemical marker to reliably predict all PTB (Menon et al., 2011; Bhat et al., 2013). The concept of utilizing multiple biochemical markers to improve labor prediction has been reported by our team (Heng et al., 2010a, 2014b; Liong et al., 2015) and others (Rizzo et al., 1998; Goldenberg et al., 2001; Kishida et al., 2003; Grenache et al., 2004; Taylor et al., 2013). Other variations of using a diagnostic panel to predict PTB include incorporating peripheral blood results, gene expression profiles (Heng et al., 2014a) as well as combining demographic, physical and behavioral parameters (e.g., cervical length or illicit drug use) (Hincz et al., 2002; Schmitz et al., 2006; Dutta and Norman, 2010; Bolt et al., 2011; Dekker et al., 2012; Fox et al., 2012; DeFranco et al., 2013).

Along with obtaining quality samples, performing rigorous clinical phenotyping and/or sub-classification of PTB, establishing reproducible analytical assays and analysing the data prudently, another significant challenge to establishing a clinical screening or diagnostic test for PTB involves the construction of a well-fitted predictive model. There is no consensus on a standard statistical approach to construct a multiple biomarker predictive model of labor. Here, we propose some important factors that should be considered when constructing a model. It should be emphasized that statistical significance does not always translate into clinical utility. Biomarkers that display a significant association with labor but may display only a small fold-change between groups and/or a large variance within groups are not likely to be clinically useful for discriminating outcomes.

### Developing a predictive model for each clinical subgroup of PTB

A model optimized to predict idiopathic spontaneous PTB may have compromised predictive capacities for PTB with known infection, PTB with multifetal gestation or preterm PROM. It may therefore be necessary to assess and consider numerous clinical parameters as confounders. Alternatively, an ideal test that could predict any possible outcome is likely to require several biomarkers that may be “unique” to each clinical subgroup.

### Determining a clinically useful prediction “window”

Different clinical presentations (e.g., at-risk asymptomatic or symptomatic women) would benefit from different sampling-to-outcome time frames or “windows.” This window will influence the type of intervention and obstetric management for each woman. For example, in women with symptoms of PTL where delivery may be imminent, a narrow prediction window of 24–48 h would be important for a clinician to decide whether or not to administer corticosteroids for fetal lung maturation and/or to transport the woman to a tertiary hospital with advanced neonatal facilities (Goldenberg, 2002; Heng et al., 2014a, 2015). Alternatively, a wide prediction window, perhaps 14–28 days would be more appropriate for asymptomatic women that will allow clinicians to place a patient under surveillance and provide therapeutic measures such as progesterone to prevent PTB.

### Selecting the most appropriate biomarkers

A reliable predictive model should be highly sensitive and specific while incorporating the fewest number of biomarkers being measured. Selecting biomarkers involved in different biochemical pathways (e.g., inflammation, ECM remodeling, oxidative stress) may value-add to a predictive test. Genetic polymorphisms associated with PTB could also be included as part of a predictive test. Similarly, one of the major advantages of proteomic methodologies is the ability to identify and compare the expression profiles of different protein isoforms. For example, the different isoforms of CSTA, FABP5, SERPINB1, and SERPINB3 displayed differing expression profiles with labor onset and the relative abundance of each isoform also varied enormously. Whilst the selection of a particular protein isoform or a genetic polymorphism may enhance a predictive test, they may be less suitable for the development of an inexpensive and rapid bedside test kit.

### Using single or multiple sampling time points

Given the large variation in biomarker concentrations between patients, the use of serial samples from the same women to determine the “change” in biomarker concentration (i.e., comparing subsequent analyte measurements against a “baseline” reference point) rather than the absolute concentration value may be more informative. Determining when this baseline measurement should be performed could be based upon fetal viability (23 weeks of gestation).

### Deciding the optimal diagnostic concentration threshold for each biomarker

Firstly, a decision should be made whether to correct the biomarker concentration to the total protein content of the CVF. This may provide a more meaningful measurement of the biomarker but would not be practical for a rapid bedside test utilizing a “dip-stick” approach similar to the fFN or phIGFBP1 tests. Furthermore, the desired sensitivity or specificity ultimately determines the concentration threshold for a positive or negative test. For example, an ideal PTB test needs to be highly sensitive where a “positive” outcome (imminent delivery) would require medical intervention to prevent labor. On the other hand, a highly specific test could be useful for predicting post-term labor as a “negative” outcome (where delivery is unlikely to occur within a defined period) would require medical intervention(s) such as induction or elective Cesarean delivery.

### Consideration of demographic, physical, and behavioral confounders

A reliable predictive test should be robust to the numerous confounding variables at play but could also be strengthened by incorporating various demographic (e.g., maternal age, ethnicity), biochemical (e.g., peripheral blood data), physical (e.g., cervical length, uterine anomaly), or behavioral (e.g., poor nutrition, illicit drug use) confounders into the predictive model (Dekker et al., 2012; Heng et al., 2014a).

Taking some of the above points into consideration, we have previously published the predictive utility of selected biomarkers using binary logistic regression (Heng et al., 2010a, 2014b; Liong et al., 2013a, 2015). We have demonstrated a superior predictive potential for labor when a combination of biochemical markers is compared with each individual biomarker. Three cytokines, IL1A, IL1B, and IL1RN were evaluated to predict spontaneous *term* labor within 3 days of sampling. As individual biomarkers, term delivery could be predicted with 52.8% sensitivity and 77.4% specificity (PPV 33.3%) using IL1A, 52.8% sensitivity and 66.1% specificity (PPV 25.0%) using IL1B and 52.8% sensitivity and 76.2% specificity (PPV 32.2%) using IL1RN. A combined cytokine model yielded 86.1% sensitivity and 91.7% specificity (PPV 68.9%) to predict labor. In this case, multiple sampling from the same woman was able to provide improved predictive efficacy even without the inclusion of demographic confounding variables (Heng et al., 2014b). In another example, ALB and GC were evaluated to predict PTL within 7 days in symptomatic women. As individual biomarkers, ALB could predict spontaneous preterm delivery with 83.3% sensitivity and 73.3% specificity (PPV 26.3%), whereas GC provided a sensitivity of 77.8% and specificity of 98.1% (PPV 77.8%). Prediction of preterm delivery within 7 days using the combined model of ALB and GC, yielded a sensitivity of 77.8%, a specificity of 100% and with 100% PPV and 98.0% NPV (Liong et al., 2015). A clinical diagnostic trial is required to test these models on a larger population to confirm these findings and further refine the predictive values. For the basis of comparison to fFN, the predictive utility of our candidate biomarkers in asymptomatic and symptomatic cohorts is summarized in Table 4.

**Table 4:** **The efficiency of putative biomarkers compared with fetal fibronectin to predict preterm labor**.

**Cervicovaginal fluid biomarker(s)**	**Time to labor (days)**	**Sensitivity**	**Specificity**	**Positive Likelihood Ratio (LR+)**	**Negative Likelihood Ratio (LR−)**	**Positive predictive value**	**Negative predictive value**	**References**
**SPONTANEOUS PRETERM LABOR (ASYMPTOMATIC)**								
fFN (meta-analysis)	7	22 (3–60)	97 (96–97)	7.3	0.80	NR	NR	Leitich et al., 1999b
fFN (meta-analysis)	14	43 (0–95)	95 (92–99)	8.6	0.60	NR	NR	Leitich et al., 1999b
fFN (meta-analysis)	28	26 (3–50)	97 (96–98)	8.7	0.76	NR	NR	Leitich et al., 1999b
GC	7	57.7	96.6	17.0	0.44	78.8	91.2	Liong et al., 2013c
GC	14	71.2	87.9	5.9	0.33	69.9	88.5	Liong et al., 2013c
IL1RN	28	57.1	97.8	26.0	0.44	72.7	95.7	Liong et al., 2013a
TRX	28	64.3	97.8	29.2	0.37	75.0	96.4	Liong et al., 2013a
IL1RN + TRX	28	64.3	97.8	29.2	0.37	75.0	96.4	Liong et al., 2013a
**THREATENED PRETERM LABOR (SYMPTOMATIC)**								
fFN (meta-analysis)	7	76 (69–82)	82 (79–84)	4.2 (3.5–5.0)	0.29 (0.22–0.38)	NR	NR	Sanchez-Ramos et al., 2009
fFN (meta-analysis)	7	75 (69–80)	79 (76–83)	3.6 (3.1–4.3)	0.31 (0.25–0.39)	NR	NR	Boots et al., 2014
fFN	7	66.7	87.9	5.5	0.38	36.4	96.2	Liong et al., 2015
IL1RN	7	88.9	50.9	1.8	0.22	13.1	98.2	Liong et al., 2015
TRX	7	55.6	79.1	2.7	0.56	20.8	94.7	Liong et al., 2015
ALB	7	83.3	73.3	3.1	0.23	26.3	97.5	Liong et al., 2015
GC	7	77.8	98.1	40.9	0.23	77.8	98.1	Liong et al., 2015
ALB + GC	7	77.8	100	>100	0.22	100	98.0	Liong et al., 2015

*NR, not reported; Range shown represents 95% confidence interval*.

## Concluding remarks

The prediction and prevention of PTB remains one of the crucial challenges facing modern obstetrics. The reliable identification of women at-risk of PTB will allow the tailoring of medical intervention and therapeutic treatments aimed at improving maternal and fetal outcomes. It is likely that the multifaceted etiology of PTB would require a multiple biomarker test. The CVF is an excellent biological fluid for the discovery of biomarkers associated with labor due to its proximity to the gestational tissues that undergo dramatic changes with advancing gestation. Proteomic approaches have enabled the discovery of novel biomarkers in the CVF involved in the physiology of labor and/or pathophysiology of PTB. These proteins have functional roles in inflammation, ECM remodeling and oxidative stress and are involved in cervical ripening and/or membrane rupture. The next phase of our work will be to validate our data with independent cohorts and integrate biochemical data with demographic, physical and behavioral information using bioinformatics approaches to develop reliable predictive models for PTB.

### Conflict of interest statement

The authors declare that the research was conducted in the absence of any commercial or financial relationships that could be construed as a potential conflict of interest.

## Footnotes

^1^0 days signifies less than 24 h before spontaneous labor onset (in the absence of symptoms and signs of labor).
